# Analysis of miRNAs Targeted Storage Regulatory Genes during Soybean Seed Development Based on Transcriptome Sequencing

**DOI:** 10.3390/genes10060408

**Published:** 2019-05-28

**Authors:** Jing-Yao Yu, Zhan-Guo Zhang, Shi-Yu Huang, Xue Han, Xin-Yu Wang, Wen-Jing Pan, Hong-Tao Qin, Hui-Dong Qi, Zhen-Gong Yin, Ke-Xin Qu, Ze-Xin Zhang, Shan-Shan Liu, Hong-Wei Jiang, Chun-Yan Liu, Zhen-Bang Hu, Xiao-Xia Wu, Qing-Shan Chen, Da-Wei Xin, Zhao-Ming Qi

**Affiliations:** 1 College of Agriculture, Northeast Agricultural University, Harbin 150030, Heilongjiang, China; Jingyaofish@163.com (J.-Y.Y.); zhanguo7907@126.com (Z.-G.Z.); 13212801125@163.com (S.-Y.H.); 15546021798@126.com (X.-Y.W.); 15645641219@163.com (W.-J.P.); Qinhongtao5565@163.com (H.-T.Q.); qihuidong1212@163.com (H.-D.Q.); yinzhengong@163.com (Z.-G.Y.); 18145648226@163.com (K.-X.Q.); Zzx781054871@163.com (Z.-X.Z.); 13263612163@163.com (S.-S.L.); j3994102@126.com (H.-W.J.); cyliucn@126.com (C.-Y.L.); hzb_net@126.com (Z.-B.H.); wuxiaoxia112233@163.com (X.-X.W.); 2 Department of Horticulture, Michigan State University, East Lansing, MI 48824, USA; hanxue5@msu.edu; 3 Green Food Research Institute in Heilongjiang Province, Harbin 150030, Heilongjiang, China; 4 Department of Plant Soil and Microbe Science, Michigan State University, East Lansing, MI 48824, USA

**Keywords:** soybean, transcriptome sequencing, miRNA, storage, target genes

## Abstract

Soybeans are an important cash crop and are widely used as a source of vegetable protein and edible oil. MicroRNAs (miRNA) are endogenous small RNA that play an important regulatory role in the evolutionarily conserved system of gene expression. In this study, we selected four lines with extreme phenotypes, as well as high or low protein and oil content, from the chromosome segment substitution line (CSSL) constructed from suinong (SN14) and ZYD00006, and planted and sampled at three stages of grain development for small RNA sequencing and expression analysis. The sequencing results revealed the expression pattern of miRNA in the materials, and predicted miRNA-targeted regulatory genes, including 1967 pairs of corresponding relationships between known-miRNA and their target genes, as well as 597 pairs of corresponding relationships between novel-miRNA and their target genes. After screening and annotating genes that were targeted for regulation, five specific genes were identified to be differentially expressed during seed development and subsequently analyzed for their regulatory relationship with miRNAs. The expression pattern of the targeted gene was verified by Real-time Quantitative PCR (RT-qPCR). Our research provides more information about the miRNA regulatory network in soybeans and further identifies useful genes that regulate storage during soy grain development, providing a theoretical basis for the regulation of soybean quality traits.

## 1. Introduction

MicroRNAs (miRNAs) are a class of endogenous small RNAs that are single-stranded RNA molecules composed of approximately 22 nucleotides [1]. Most miRNAs exist in the genome as single copies, multiple copies or clusters of genes [2]. Together with short interfering RNAs (siRNAs), miRNAs are a key part of the evolutionarily conserved system of RNA genes and play an important regulatory role in plants and animals [3]. MicroRNA database (MiRBase) (http://www.mirbase.org/) is a searchable database of published miRNA sequences and annotations, containing miRNA information for a variety of plants and animals [4]. With the emergence of soybean genome-scale experiments and a large amount of biological data, the Soybean Knowledge Base, a comprehensive network resource of soybean genomics, has been constructed. The platform manages and integrates genomics, transcriptomics, proteomics and metabolomics data and analyzes gene function and annotation of biological pathways, including four entities, genes, microRNAs, metabolism and single nucleotide polymorphisms (SNPs) [5]. Studies have shown that miRNAs negatively regulate their target genes. A variety of adverse conditions can induce the production of miRNA, whose purpose is to regulate the target genes by directing the degradation of the mRNA of the target gene and preventing translation and finally adapting to the adverse environment through morphological or physiological changes. Each miRNA can have multiple target genes, and several miRNAs can also regulate the same gene. Many studies are based on transcriptome sequencing to analyze microRNA gene regulation.

Currently, an increasing number of scientific studies have penetrated the field of miRNA research, it plays a key and diverse role in symbiotic nitrogen fixation (SNF), nutrient acquisition and plant development [6] and more studies have found that the regulation of miRNA is related to adversity; however, only a few of these studies have investigated soybean quality traits. The storage of soybean cotyledons directly affects soybean seed yield and quality. By sequencing miRNAs in soybean cotyledons, Goettel et al. found that a total of 304 miRNA genes were expressed in soybean cotyledons and predicted 1910 of their targeted genes. Then, Goettel et al. integrated these genes with their miRNAs into a complex miRNA network that regulates the cotyledon biological pathway and reveals the evolutionary pathways of soybean miR15/49 in soybean cotyledons [7]. To resolve miRNA-directed gene regulation in soybean seed development, Shamimuzzaman and Vodkin (2012) examined miRNA-directed gene regulation in soybean seed development by transcriptome sequencing using degenerate set-sequencing and detecting the cleavage of miRNA targets. A total of 183 different targets of 53 known soybean miRNAs were identified [8]. Soybean lipid metabolism may be regulated by complex noncoding networks. By focusing on the genes involved in lipid metabolism, a large number of endogenous target mimics (eTMs) and phased siRNA-generated loci (PHASs) were found. The genes related to lipid metabolism may be regulated by 28 miRNAs, 9 of which may be further regulated by many eTMs with evidence of expression [9].

In this study, based on a chromosome segment substitution line (CSSL) population previously crossed with SN14 and ZYD00006, four samples with extreme phenotypes, high or low protein and oil content, and their recurrent parents were examined in three seed development stages by RNA sequencing and expression analysis. The expression patterns of target genes and miRNAs were analyzed According to the analysis, important target genes associated with the storage composition during the development of soybean seeds were identified, providing a basic molecular mechanism for soybean breeding.

## 2. Materials and Methods

### 2.1. Plant Growth and Sample Selection

**Figure 1:**
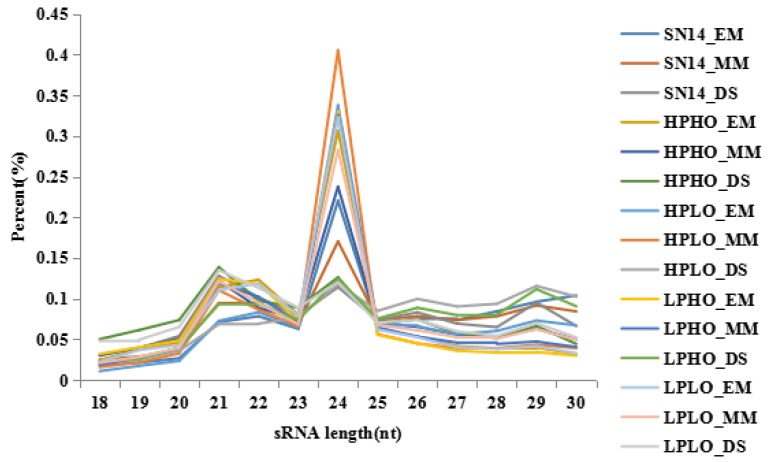
Statistics of small RNA strand length distribution. Small RNAs with materials [Suinong 14 (SN14), High Protein Low Oil (HPLO), Low Protein High Oil (LPHO), Low Protein Low Oil (LPLO), High Protein High Oil (HPHO)] in three stages [Early Maturity Stage (EM), mid seed maturity (MM), dry seed stage (DS)] are shown in different color lines.

Previously, the laboratory constructed the chromosome segment substitution line (CSSL) which was crossed with cultivar SN14 and wild soybean ZYD0006, and constructed with SN14 as the recurrent parent. We use SN14 to hybridize with ZYD00006, and then BC_3_F_1_ was obtained after three years of backcrossing and then self-crossing for many years. Now, the genetic background of each progeny of this population was SN14, and only a small part of the introduced fragment was wild soybean ZYD00006 [10]. Four lines, high protein high oil (HPHO), low protein low oil (LPLO), low protein high oil (LPHO), and high protein low oil (HPLO), with the phenotypes of protein and oil content in the top 10% and the bottom 10% of the CSSL population distribution in 2014–2016 were selected, and there was no significant difference in other traits from SN14, as illustrated in Figure 1a by Qi et al. (2018) [11]. Samples were taken from different pods of different plants in three representative stages of seed size standard (EM, MM, DS) during the development of the soybean seed in order to eliminate individual differences. The EM stage is Early Maturity Stage, the MM stage refers to mid seed maturity and the DS stage means dry seed stage. The criteria for each stage are detailed in Supplementary Table S1 [12]. The interval between every two samplings was approximately 20 days from July to the end of September. Samples were snap frozen in liquid nitrogen and stored at −80 ℃. Details of the construction of the CSSLs, plant growth and each sampling stage are described in Qi et al. (2018) [11].

### 2.2. Small RNA Extraction and Library Construction

Soybean seeds stored at −80 °C were ground under liquid nitrogen conditions. Total RNA was extracted using TRIzol Reagent (Invitrogen, Carlsbad, CA, USA, Cat no.15596-026) for RNA-seq and small RNA-seq [13]. A NanoDrop instrument was used to determine RNA integrity and purity and an OD value between 1.8 and 2.2 was required. Electrophoresis detection of 28S:18S was at least 1.5, and the total RNA concentration was not less than 750 ng/μg. Small RNA was isolated and purified from total RNA, and small RNAs of 18-30 nt in length were selected. Using the Small RNA Sample Prep Kit, 3′ and 5′ linkers were connected to the samples that met the qualifications, and subsequently subjected to reverse transcription amplification. Libraries were constructed using the Illumina TruSeq kit (RS-200-0012; Illumina Inc., San Diego, CA, USA). Then, Reverse Transcription PCR (RT-PCR), purification of the small RNA library, library detection (electrophoresis detection, NanoDrop and Agilent Technologies 2100 analyzer) and PCR amplification were performed in sequence. The target-sized fragment was separated by PAGE gel electrophoresis. Finally, high-throughput sequencing was performed on the Illumina platform (Illumina HiSeq 4000 SE 150).

### 2.3. Small RNA Data Analysis

The raw data obtained by high-throughput sequencing (such as the Illumina sequencing platform) were converted into the original sequencing sequences by base calling analysis. For the resulting sequences and raw data, the process of removing low quality, linked, and contaminated reads was accomplished by the preliminary processing of the data to obtain target sequences with high-quality and clean reads. To control the quality of raw data in the high-throughput sequencing, the FastQC Toolkit was used [14]. The results were stored in a FASTQ (abbreviated as fq) file format containing sequence information for the sequencing sequence and its corresponding sequencing quality information. For the clean reads of each sample, the small RNAs within a certain length range were screened for subsequent analysis and a length distribution map for the small RNA sequence was made. The SOAP (V2.20) software was used to map the small RNAs to the reference genome of soybean (*Glycine max* Wm82.a2.v1) and to analyze the expression and distribution of the small RNAs on the reference genome [15]. The clean reads of each sample were compared with the Repeat sequence, GenBank, Rfam and miRBase databases and were fully classified. The possible rRNA, scRNA, snoRNA, snRNA and tRNA reads were found and removed as much as possible. The miRNAs were identified by comparing the clean reads of the small RNAs of the SN14 and CSSL lines with the miRNAs of soybean in the miRBase database (miRBase 21.0, http://www.mirbase.org/) [4,16].

In this study, the expression levels of miRNAs in each sample of SN14 and the 4 CSSL introduced lines with extreme phenotypes were statistically analyzed. The TPM (tag per million) algorithm was used to normalize the expression of each sample. The TPM normalization formula is:

(1) $$\mathrm{TPM}=\frac{\mathrm{Readcount}\;\times 1,000,000}{\mathrm{MappedReads}}$$

where readcount represents the number of reads aligned to a miRNA and MappedReads represents the number of reads of all miRNAs.

The raw data have been uploaded to the BIG Data Centre (Beijing Institute of Genomics, Chinese Academy of Sciences, http://bigd.big.ac.cn/gsub/, project number: PRJCA001149, data submission ID: CRA001271).

### 2.4. Targeted Gene Prediction

By using the mirBase database (http://www.mirbase.org/), the most commonly used miRNA database, predictions for the miRNA-targeted genes were made [16]. For the most part, computational methods rely on first detecting potential binding sites (with a large degree of complementarity to the miRNA), followed by filtering out those sites that do not appear to be conserved in multiple species. Its core algorithm assigns *p*-values to individual miRNA–target binding sites, multiple sites in a single UTR, and sites that appear to be conserved in multiple species based on robust statistical models [17]. The core prediction of another calculation method is internally generated using the miRanda algorithm (v3.0) [18]. The provision of a *p*-value for each miRNA–target assignment allows the user to assess the confidence in the prediction. A three-stage method for identifying target sites from sequence information based on the work of Enright et al [18]. wherein sequence matching first assesses whether two sequences are complementary and possibly combined; free energy calculation (thermodynamics) is used to estimate the energy of this physical interaction and evolutionary protection as an information filter.

### 2.5. Differentially Expressed miRNA and Gene Analysis

In this study, differential expression of miRNAs and genes were analyzed by using the DEGseq software package for normalizing expression levels and performing differential expression analysis based on negative binomial distributions [19]. Each sample was mixed sampling, the known differentially expressed miRNAs and genes were detected by using and as the screening criteria.

### 2.6. Verification of Candidate Genes by RT-qPCR

Extracting RNA from soybean seed development stage (EM, MM, DS) samples by Trizol, genomic DNA was removed using Vazyme’s 4 × gDNA wiper Mix, and 5 × HiScript II qRT SuperMix II reverse-transcribed RNA into a single-stranded cDNA. The Real-time Quantitative PCR (RT-qPCR) experiment was performed using a 2 x ChamQ Universal SYBR qPCR Master Mix kit on the LightCycler480 System (Roche). Three biological replicates were performed for each RT-qPCR experiment, and the relative expression of the soybean seed candidate genes were calculated according to:

(2) $$\mathrm{Relative}\;\mathrm{Expression}=2^{∆Ct},\;\;\left[∆Ct=Ct\left(GmActin4\right)-Ct\left(targetgenes\right)\right]$$

The specific primer for the gene was designed by the software Primer 5, and the internal reference gene was *GmActin4* [20].

### 2.7. AgriGO Analysis

AgriGO (http://bioinfo.cau.edu.cn/agriGO/analysis.php) was used to perform enrichment analysis on the Gene Ontology (GO) number of the genes, and the gene classifications were clustered into several GO annotation categories [21]. We focused on the target genes related to their corresponding miRNAs. By analyzing the miRNA-related target genes by AgriGO, the target genes are classified according to GO annotations and narrow the range of the target genes associated with seed storage materials.

## 3. Results

### 3.1. Global Analysis of Small RNA-Seq Data

The raw data of SN14 and four CSSL samples with extreme phenotypes of high protein high oil (HPHO), high protein low oil (HPLO), low protein high oil (LPHO) and low protein low oil (LPLO), are shown in Table S2. The base quality value is an important indicator of the quality of measurement, the Q20 values for all sequencing raw data were above 95%, and the Q30 values were mostly above 90%, which is in accordance with sequencing standards. The reads quality score was improved by additional filtering, and the reads obtained by sequencing were filtered to obtain the clean reads. Clean reads of each sample were screened for small RNAs of a certain length for subsequent analysis. For the clean reads of each sample, the length distribution map of the small RNA sequence showed that the length of the small RNAs was 18~30 nt, and the length of the miRNAs was primarily 21 or 22 nt. The length distribution of the small RNAs measured in this study is shown in Figure 1. Consistent with the field, the sequencing results obtained in this study are of good quality and can be used for subsequent analysis. The small RNAs were mapped to the soybean reference genome (*Glycine max* Wm82.a2. v1) by SOAP (V2.20) software, and the expression and distribution of the small RNAs on the reference genome were analyzed. Total sRNAs (total clean reads) ranged from 13,942,845 to 22,962,665, with a total alignment ranging from 60.36% to 70.16%. The expression level of each sample was normalized as TPM (tag per million), and miRNA and their target genes were also predicted, including 2564 pairs (Table S3). The expression levels of some miRNAs decreased from EM to MM, and increased from MM to DS, that is, the expression level was lower in the MM stage, and the expression levels in the EM and DS stages were higher when compared with those in MM stage. Others behaved in the opposite direction. Compared to the expression of the target genes, the miRNA negatively regulated the target genes.

### 3.2. Differential Expression Analysis of Known miRNA

**Figure 2:**
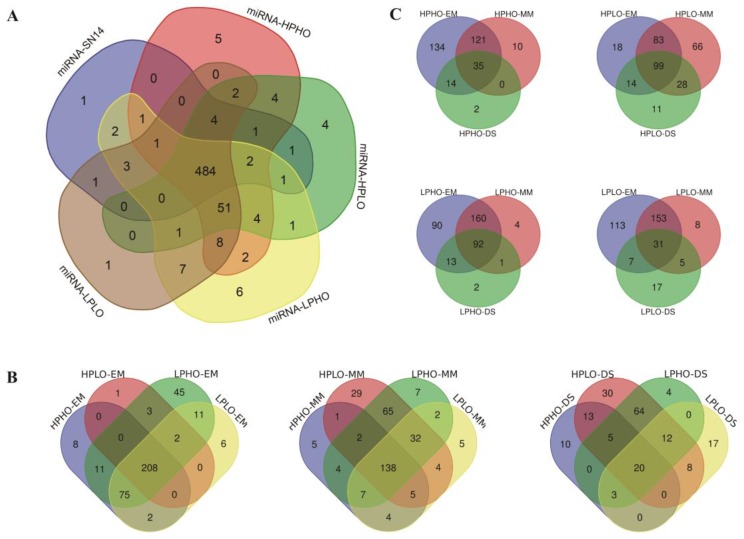
Venn diagram that specifically expressed MicroRNA (miRNA) in four lines (HPHO, HPLO, LPHO, LPLO) at the EM, MM, DS stage. (**A**) Expression of miRNAs in various materials. (**B**) Differential expression of known miRNA at EM, MM, DS stage (**C**) Differential expression of known miRNA for each extreme material.

The known miRNAs in SN14 and the four CSSL samples with extreme phenotypes were summarized, and a total of 606 known miRNAs were expressed in the samples. Specifically, there were 506 known miRNAs in SN14 belonging to 79 families; 569 known miRNAs in HPHO belonging to 81 families; 568 known miRNAs in HPLO belonging to 82 families; 582 known miRNAs in LPHO belonging to 82 families and 571 known miRNAs in LPLO belonging to 81 families. There were 484 known miRNAs expressed in all the samples. The specific level of expression for the known miRNAs in each sample was shown in Figure 2A. A known miRNA specifically expressed in SN14 was gma-miR1516a-3p. Five known miRNAs specifically expressed in HPHO material, namely gma-miR1516d and gma-miR172b-5p, gma-miR1521b, gma-miR5667-5p and gma-miR9764. There were four known miRNAs (gma-miR9739, gma-miR5674b, gma-miR5674a and gma-miR1526) specifically expressed in HPLO materials. Six known miRNAs specifically expressed in LPHO were gma-miR5371-5p, gma-miR169c, gma-miR171h, gma-miR4348c, gma-miR172g and gma-miR1517. Only one known miRNA specifically expressed in LPLO material: gma-miR4368a.

Differential expression analysis was performed by the DEGseq software package to quantify the difference in the expression of known miRNAs among the four CSSL samples with extreme phenotypes and the SN14 samples at the same stage using and as standers (Table S4). The analysis considered significantly differentially expressed miRNAs regarding the two different attributes of sample type and developmental stage. First, the distribution of known miRNAs with significant differences among the various samples during each of the three stages EM, MM, and DS was shown in Figure 2B. Based on the known miRNAs of the three stages, the known miRNAs of the four CSSLs belong to several families that more than the known miRNAs of SN14, such as Mir4372, Mir4374, Mir5225, Mir5559 and Mir5674. As compared to the extreme materials of any two CSSLs, there was a difference in the family to which the known miRNAs belong, which may be related to the different quality traits of the materials. Second, the distribution of known miRNAs with significant differences among three stages (EM, MM, DS) in each of the four samples with extreme phenotypes (HPHO, HPLO, LPLO, LPHO) was shown in Figure 2C.

**Figure 3:**
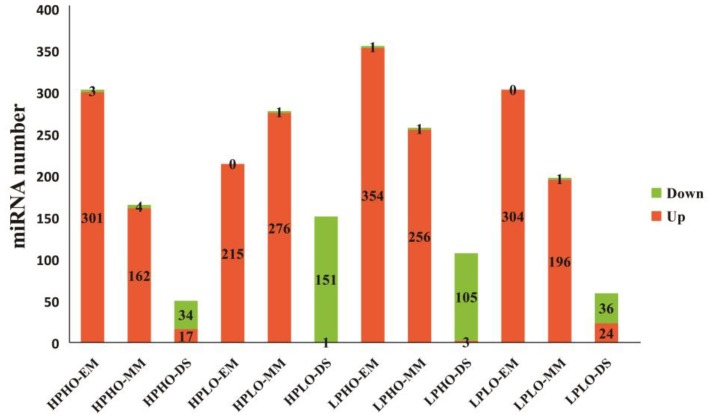
The number of differentially expressed up-regulated miRNAs and down-regulated miRNAs in four lines (HPLO, LPHO, HPHO, LPLO) compared with the control at three stages (EM, MM, DS). Up-regulated miRNAs are represented in orange and down-regulated miRNAs are represented in green.

The number of up-regulated miRNAs among the differentially expressed miRNAs in the SN14 samples and four CSSL samples with extreme phenotypes at various stages was shown in Figure 3. As seen from Figure 3, for each material, the most significantly differentially expressed known miRNAs in the EM and MM stages showed upregulation, and only the significantly impacted unknown miRNAs showed downregulation; however, during the DS stage, the number of known miRNAs exhibiting downregulation was significantly higher than that of the number upregulated known miRNAs. This pattern was especially evident in the HPLO and LPHO samples, which showed exactly the opposite results in the DS stage than in the first two stages.

**Figure 4:**
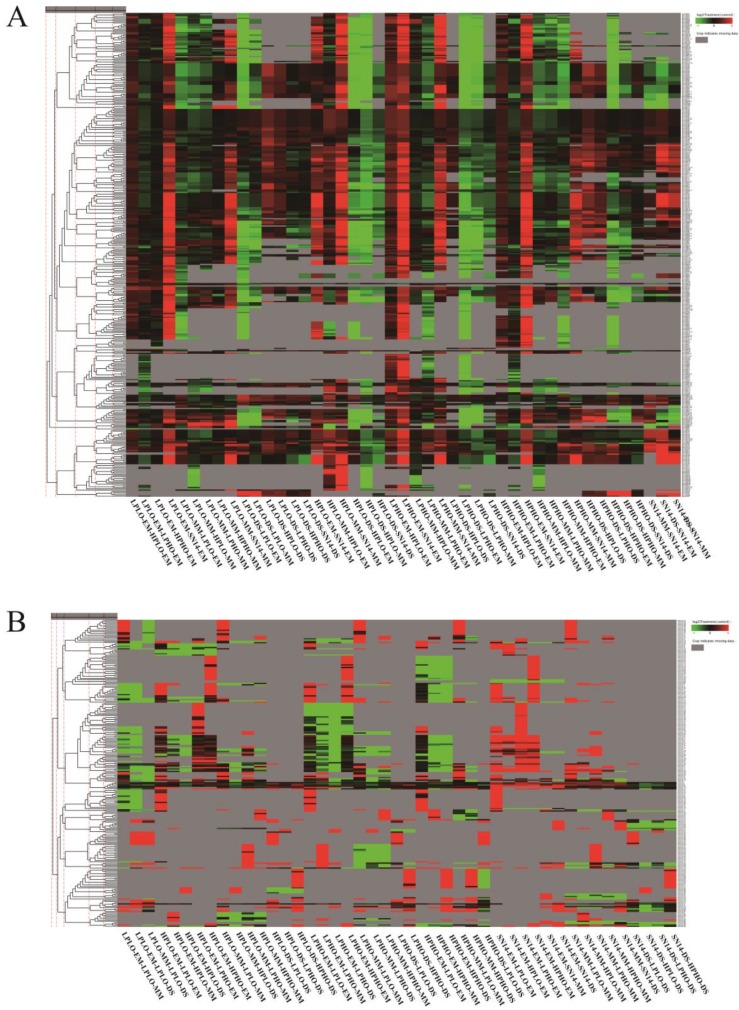
Cluster analysis of miRNAs with similar expression patterns. The heatmap showed miRNAs for each cluster that were significantly expressed in control, HPHO, HPLO, HOLP and LPLO at the EM, MM and DS stages, respectively. The Samples with miRNA expression levels higher than control material showed color red, the Samples with miRNA expression levels lower than control material showed color green, and the color gray indicates that the miRNA is not expressed in at least one sample. (**A**) Cluster analysis of known miRNAs, (**B**) Cluster analysis of novel miRNAs.

The known miRNAs in all materials belonged to 84 miRNA families, however, there was no known miRNAs belonging to the Mir167 family found in the samples of the SN14 and 4 CSSLs. Cluster analysis grouped miRNAs with similar expression patterns (Figure 4). The Mir394 family of miRNAs showed a significant upregulation at the MM stage of the four materials and a significant downregulation at the DS stage, especially in the HPLO and LPHO samples. The miRNAs from the Mir164 family showed significant differences in the four materials at different stages. At the EM stage, the Mir164 family miRNAs were expressed at higher levels in HPHO and LPHO than in the other two sample types. At the MM stage, the expression levels of the Mir164 family miRNAs in HPLO and LPHO were high, and the expression level of these miRNAs in the four samples was higher than that of the control sample SN14 in the EM and MM stages. However, compared to the DS stage, the expression levels of the miRNAs in each sample in the EM and MM stages were lower than that of the control SN14, and the expression levels of LPHO and HPLO were lower. The expression levels of the Mir164 family miRNA target genes *Glyma.17G101500*, *Glyma.06G195500* and *Glyma.05G025500* were lower than the levels in the control SN14 in the MM stage. The expression of the target gene *Glyma.15G254000* in the HPLO sample was extremely high compared with the control material SN14 in the MM stage. The miRNA expression of the Mir166 family had a similar pattern. The targeting gene of the Mir166 family, *Glyma.09G023600,* also showed differential expression levels in the different phenotypes at different stages.

### 3.3. miRNA Regulation of Target Genes

The regulation of differentially significant target genes by miRNAs can be divided into several different modes: one gene is regulated by multiple miRNAs of the same family; one miRNA simultaneously regulates multiple different genes, and these genes can be seen to have the same function by annotation; one gene is regulated by only one miRNA.

For HPHO, there were 316 differentially expressed miRNAs in three stages, of which 225 known miRNAs predicted 496 target genes. For HPLO, there were 319 differentially expressed miRNAs in three stages, of which 240 known miRNAs predicted 537 target genes, and 456 of these target genes have higher expression levels. There were 362 differentially expressed miRNAs in LPHO in three stages, of which 248 known miRNAs predicted 558 target genes, and 470 of these target genes were highly expressed compared with the same stage of control. Similarly, for LPLO, there were 334 differentially expressed known miRNAs in three stages, of which 235 known miRNAs predicted 525 target genes, and 432 of these target genes were highly expressed compared with SN14.

A total of 597 pairs of corresponding relationships between novel-miRNA and their target genes were also obtained. For novel-miRNA, it was also expressed at different stages of each material, but not fully expressed. Most of the novel-miRNAs were expressed in the EM stage, and some novel-miRNAs were expressed in the late stage of seed development, and only a few were expressed in various stages. Its expression level was lower than that of known-miRNA. However, novel-mir-220 was highly expressed in the EM, MM stage, targeting the regulatory gene Glyma.13G193300. Novel-mir-119, novel-mir-194, novel-mir-2, novel-mir-246, novel-mir-31 and novel-mir-66 were expressed in all stages. Among them, novel-mir-2 and novel-mir-246 were more prominent because of thier high expression level. Novel-mir-2 was targeted to Glyma.01G051600, which was annotated as MYB domain protein 16.

### 3.4. Analysis of the Regulation of Target Genes by Differentially Expressed Known miRNAs

Investigating the target genes of the miRNAs revealed a complex regulatory relationship (Table S3). We selected both miRNAs and their target genes highly specific expressed, that is, genes and miRNAs that were differentially expressed both at different stages of seed development and in different phenotypes. The expression of the gene was obtained by sequencing the transcriptome from Qi (2018) [11] (Table S5). The functions of these genes were annotated in order to screen for genes involved in the growth and development of soybean seeds, especially proteins, fatty acids or sugars.

*Glyma.13G035200* and *Glyma.14G156400* was regulated by Gma-miR2119, both of which are annotated as an alcohol dehydrogenase 1, associated with lipids. This dehydrogenase oxidizes the ω2-hydroxy fatty acid to the corresponding oxidized fatty acid but has no significant activity on the ω1-hydroxy fatty acid [22]. From EM to DS, gma-miR2119 showed a decrease in relative expression in the HPHO, LPHO, and HPLO lines, and compared with SN14, the expression level of this miRNA was higher. In the LPLO line, the expression level was higher than the levels in the SN14 samples in the EM stage and was lower in the MM stage. In the HPLO and LPHO lines, the relative expression of *Glyma.13G035200* increased from the EM stage to the MM stage and decreased to the DS stage. In the HPHO line, the relative expression level of this gene increased throughout the developmental stages. In the LPLO line, the relative expression of this gene decreased from EM to MM and increased from MM to DS, where it reached the highest point. The relative expression of *Glyma.14G156400* decreased first after the EM stage and then increased by the DS stage in the HPHO line. However, in the HPLO and LPHO lines, the opposite expression pattern occurred. The relationship between the expression of these two genes and the miRNA all showed negative regulation.

Another lipid-related gene is *Glyma.09G023600*, which is annotated as a homeobox-leucine zipper family protein/lipid-binding START domain-containing protein. This START domain regulated the fatty acid metabolism of proteins in a lipid-dependent manner and played a role in lipid regulation of intracellular signaling pathways in animals and plants [23,24]. *Glyma.09G023600* is regulated by 20 miRNAs in the Mir166 family. In each phenotypic line, the gene had a negative correlation with the miRNAs, especially in the HPLO line.

**Figure 5:**
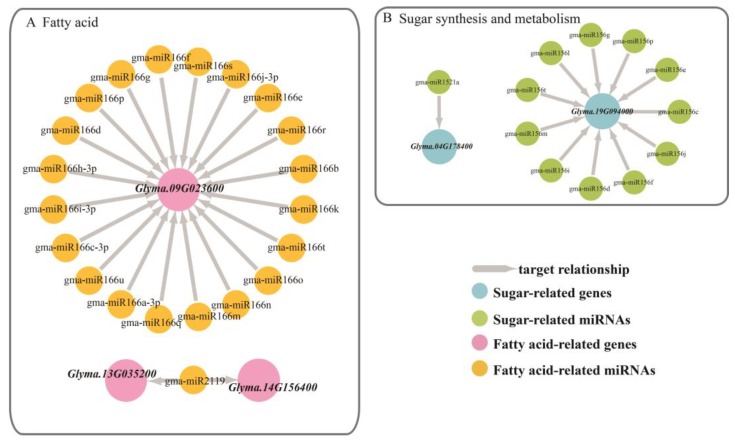
Regulatory network relationships between differentially expressed miRNAs and their targeted genes related to storage materials. The arrow represented targeted regulation. (**A**) The miRNA regulatory network targets genes associated with fatty acids. Yellow circle represented fatty acid-related miRNAs and pink circle represented fatty acid-related genes. (**B**) The miRNA regulatory network targets genes associated with sugar synthesis and metabolism. Green circle represented sugar-related miRNAs and blue circle represented sugar-related genes.

**Figure 6:**
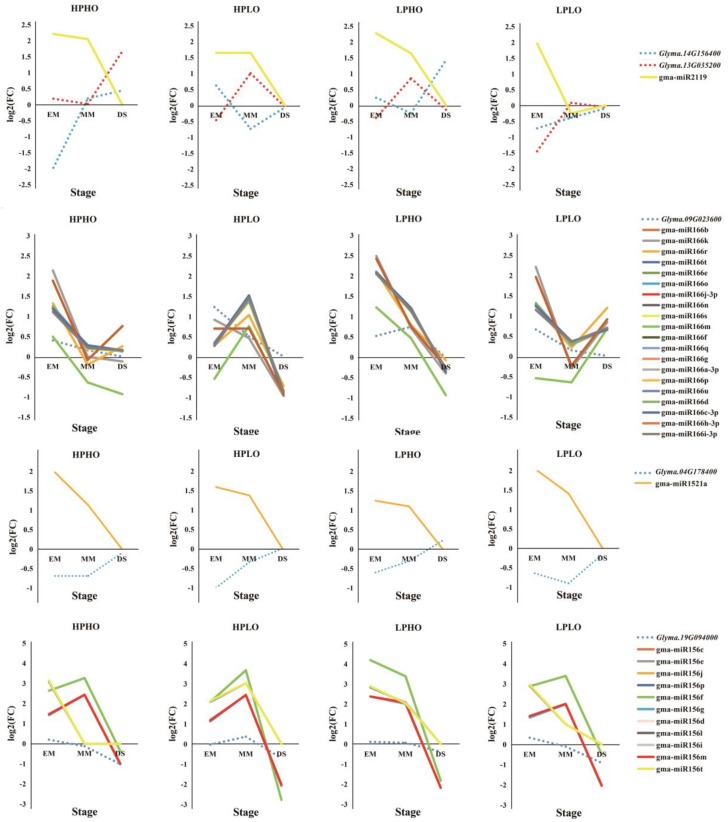
Expression relationship between miRNA and its target gene. The dotted line represented the trend of the gene expression in each stage. The solid line represented the trend of miRNA expression in various stages. The *X*-axis represents the developmental stage of the seed, and the *Y*-axis represents the relative expression level.

*Glyma.04G178400* targeted Gma-miR1521a belonging to the mir1512 family, which was annotated as an ADP-glucose pyrophosphorylase family protein. This enzyme mainly regulates the synthesis of glycogen and starch by extending the α-1,4-glycosidic chain at the ADP-glucose synthesis level by using ADP-glucose as a glucosyl donor [25], which plays a key role in regulating starch biosynthesis in cereal seeds and may be the most important determinant of seed bank strength [26]. From the EM stage to the DS stage, the relative expression of *Glyma.04G178400* increased, while the relative expression of gma-miR1521a decreased, showing a significant negative correlation. The gene *Glyma.19G094000* was co-targeted by 11 miRNAs that belong to the mir156 family and were annotated to be involved in sugar synthesis and metabolism. The SQUAMOSA promoter-binding protein-like (SPL) gene has been shown to play important roles in plant growth and development. Chen et al. (2010) noted that SPL plays a role in the regulation of other transcription factors and may also be involved in basic metabolic processes, such as glucose metabolism, according to the preliminary network and interaction group analysis. In the targeted relationship between this gene and miRNAs, the relative expression of miRNA far exceeded the relative expression of the gene [27] (Figure 5 and Figure 6).

### 3.5. Expression Analysis of Candidate Target Genes by RT-qPCR

**Figure 7:**
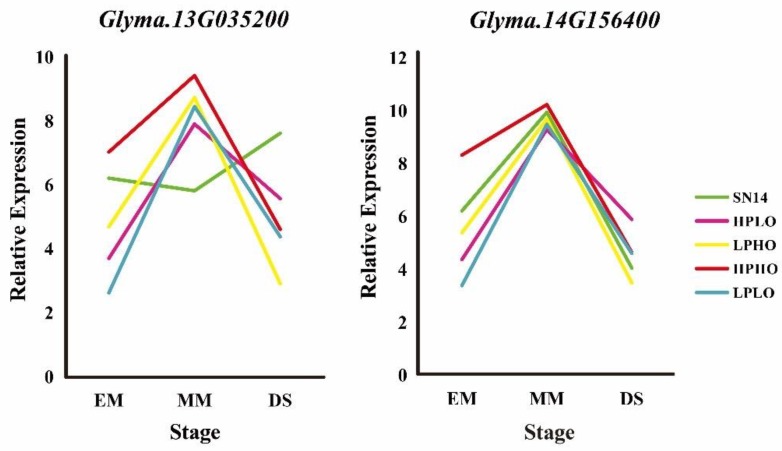
Expression analysis of miRNA targeting genes. The *X*-axis represents the developmental stage of the seed, and the *Y*-axis represents the relative expression level. Different colored lines represent different materials.

Organ expression level information of the five candidate genes was obtained from the phytozome website, wherein *Glyma.14G156400* showed a low expression level in the seed. *Glyma.04G178400* was annotated with sugar and the results showed that the gene expression pattern was not consistent with the four extreme materials in RT-qPCR. The expression of *Glyma.09G023600* in the stem was high, and the results of RT-qPCR showed that the expression level of the gene was not obvious during seed development stages. *Glyma.13G035200* and *Glyma.14G156400* have high expression levels in seeds. During seed development stages, from EM to MM, the expression of these genes increases, and from MM to DS decreases, which is consistent with the expression pattern of previously studied genes. The expression level of these genes in high oil content materials (HPHO, LPHO) is higher than that of low oil content materials (HPLO, LPLO) (Figure 7).

### 3.6. Agrigo Enrichment Analysis of Predicted Target Genes for miRNAs

A total of 2564 pairs of effective miRNA-gene targeting relationships were obtained, including 1967 pairs of relationships between known miRNA and their target genes, and 597 pairs of novel miRNAs matching target genes were found.

**Figure 8:**
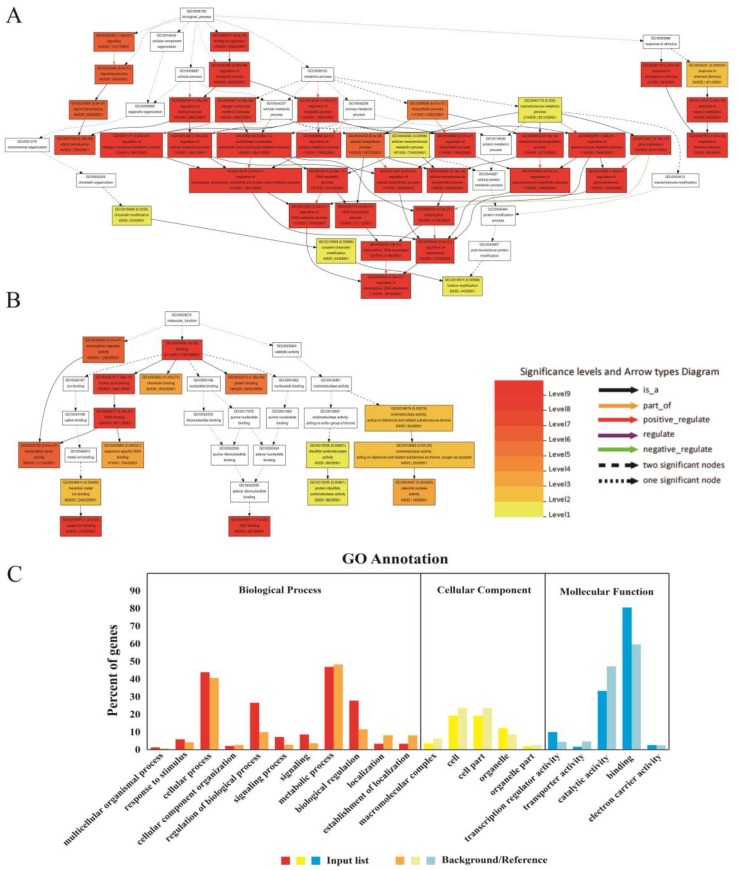
Agrigo Enrichment Analysis of target genes. AgriGO enrichment at false discovery rate (FDR) <0.01 level, classification of genes by GO annotation enrichment, resulting in three major types of biological processes, cellular components and molecular functions. (**A**) Biological process. (**B**) Molecular function. (**C**) GO analysis of targets of known and new miRNAs in this study. The dark column represents the enrichment of miRNA target genes in the GO terminology. Light column represents the percentage of total annotated soybean genes mapped to GO terms.

GO enrichment analysis of miRNA target genes by the AgriGO website revealed that most of the target genes were enriched into biological process and molecular function, and a small number of target genes were related to cellular component. In terms of biological processes, many GOs corresponding to genes showed extremely high levels of significance. More than 40% of genes were enriched in GO: 0009877 (cellular process) and GO: 0008152 (metabolic process). In addition, more than 20 percent of genes were enriched in GO: 0050789 (regulation of biological process) and GO: 0065007 (biological regulation). In terms of molecular function, more than 80 percent of the genes were annotated as binding, and more than 30% of the genes were annotated as GO: 0003824 (catalytic activity) (Figure 8).

## 4. Discussion

With the development of genomics, transcriptomics, proteomics and metabolomics have emerged. Among them, transcriptomics is the most widely used technology [28]. The transcriptome mainly includes mRNA and noncoding RNA [29] and is the basis of gene function and structure studies. For transcriptomics research, transcriptome sequencing is a powerful tool for the further study of transcriptomes, and its applications are becoming more widespread. Transcriptome sequencing can detect the overall transcriptional activity of arbitrary species at the single nucleotide level, identify unknown transcripts and rare transcripts while analyzing the structure and expression levels of transcripts and can provide more comprehensive transcriptome information. Soybean molecular biology research could be improved by matching the transcriptome-sequenced data to the reference genome. In recent years, an increasing number of articles have been published that used RNA-seq technology, and many of them were related to soybean [30]. As regulators of gene expression, miRNAs are widely found in animals and plants. Functional elucidation and target analysis of the known and novel miRNAs could yield more clues to the different regulations of gene expression between species. This could provide additional clues for the different regulation of gene expression between species by the functional annotation of candidate genes and a targeted analysis of miRNAs [1]. In our study, through RNA-seq, we identified 2564 specific relationships between genes and miRNAs and five hub genes were found.

CSSLs are ideal samples for genetic research at the genetic level and have been widely used in various plant species, such as rice [31], maize [32], wheat [33] however, few have been used in soybean. In this study, 194 soybean CSSL populations were constructed from wild soybean ZYD00006 and Chinese cultivar SN14 [8]. The CSSL population with wild species as donor parents is rich in genetic resources have only few differences in genetic background and is the preferred material for the study of complex quantitative genetic traits. The four CSSL lines selected in this study only showed significant differences in protein and oil content, and there was no significant difference in other phenotypes. This design narrowed the range of differentially expressed genes, allowing us to accurately mine the fragments and important candidate genes. In this study, several important candidate genes for seed oil and protein content were identified based on the CSSL population.

**Figure 9:**
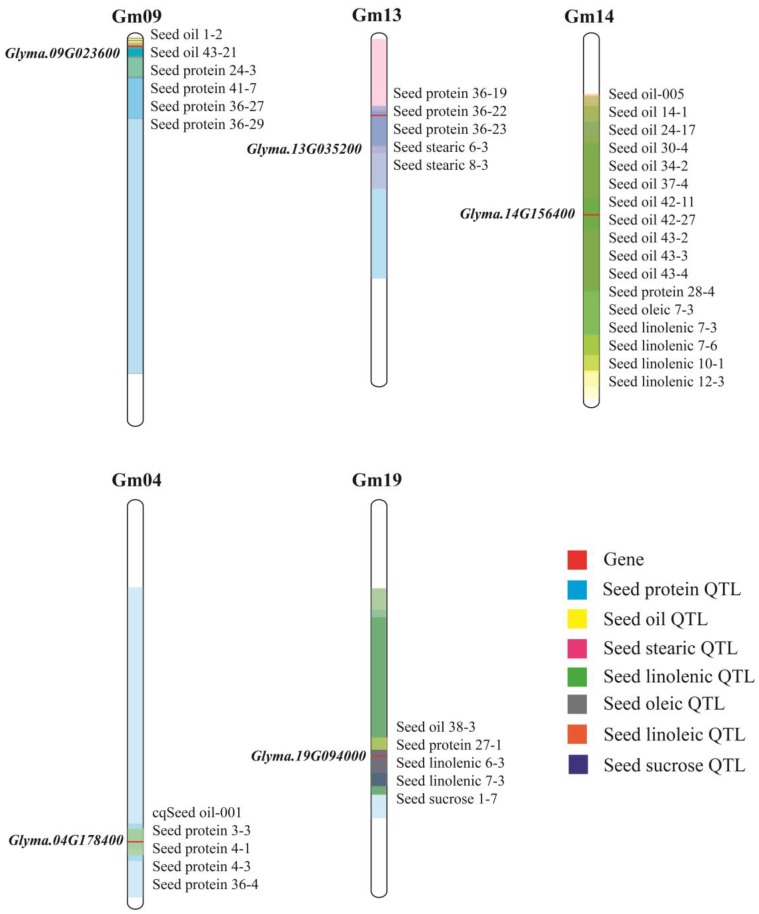
Quantitative trait locus (QTL) validation of candidate genes. Different color modules represent different types of QTL, light blue for seed protein QTL, yellow for seed oil QTL, pink for seed stearic QTL, green for seed linolenic QTL, gray for seed oleic QTL, orange for seed linoleic QTL and dark blue for seed sucrose QTL. The genes are marked in red. The transparency of the color is increased so that the overlapping area is easier to distinguish.

Published quantitative trait locus (QTL) information was collected, including 230 seed oi QTLs, 313 seed protein QTLs, 430 fatty acid component QTLs, and 57 seed sugar QTLs [11]. *Glyma.09G023600* was in the common interval of Seed oil 1-2, Seed oil 43-21, Seed protein 24-3, Seed protein 36-27, Seed protein 36-29. *Glyma.13G035200* was in the common interval of Seed oil 24-25, Seed protein 36-19, Seed protein 36-22, Seed protein 36-23, Seed stearic 6-3, Seed stearic 8-3. *Glyma.14G156400* was located in multiple pre-positioned QTL intervals about 11 oil QTLs, one protein QTL and five fatty acid component QTLs. *Glyma.04G178400* and *Glyma.19G094000* were contained in five QTLs respectively (Figure 9). Meta-analysis is a scientific quantitative method that can integrate and analyze results of similar studies to reveal the consistency of some research [34,35]. Currently, meta-analysis has been improved to offer a reliable and effective new analytical method for genetic breeding, and combining meta-analysis with research is a very meaningful approach [20,36]. From the Meta-QTL mentioned in Qi (2018) [11], the genes *Glyma.13G035200* and *Glyma.14G156400* related to oil are contained in MQTLSA-2 and MQTLLNA-1, respectively. This is more powerful proof that these genes regulate oil accumulation during soybean seed development. Although *Glyma.13G035200* and *Glyma.14G156400* were also determined to be related to alcohol dehydrogenase [37], they showed a strong specific response to flooding [38]. However, we screened these genes in high or low protein and oil lines by high-throughput transcriptome sequencing and found that they were related to the accumulation of seed storage materials and to seed growth and development.

WGCNA is an algorithm that can effectively search for important hub genes, cluster modules, regulatory networks, corresponding traits correlations, and modules and different trait relationships. Combined with the transcriptome analysis [11], 58 different modules were identified by 47,265 genes based on the pairwise correlation of gene expression in all samples. The fatty acid related genes are enriched in seven modules, and the storage protein related genes are enriched in eight modules. The genes identified by the miRNA-regulated are in the key networks. *Glyma.13G035200* is in the co-expression network of salmon module related storage protein. *Glyma.04G178400* and *Glyma.14G156400* are in the dark orange module associated with the fatty acid. In addition, the black module is also an important fatty acid related module, and *Glyma.09G023600* is included. *Glyma.19G094000* is involved in purple module associated with seed storage protein.

RT-qPCR is a method to verify the expression pattern of genes. In our study, the results of RT-qPCR showed that the genes *Glyma.13G035200* and *Glyma.14G156400* were consistent with the expression pattern of protein and oil genes and their expression level in high oil materials was higher than in low oil materials.

Although many studies on the identification of genes and miRNAs have been published, more previous studies are based on soybean stress resistance or nodulation, and there are few studies related to seed growth and the development of quality traits.

In this study, a transcriptome sequencing analysis of soybean seed development stages were carried out and we focused on microRNAs and their target genes related to seed storage materials. The parents SN14 and the chromosome segment substitution line (CSSL) built with SN14 and ZYD00006 screened four materials with obvious and stable differences in protein and oil phenotype, were selected as plant materials. According to the sequencing results, we focused on the miRNAs specifically expressed in different stages of each material and found their targeted genes. The genes associated with soybean seed storage materials were screened by the annotation of genes, and these targeted genes were verified by RT-qPCR. Finally, two genes (*Glyma.13G035200* and *Glyma.14G156400*) that promote the accumulation of soybean oil content were identified. These two genes were regulated by miRNAs and annotated as alcohol dehydrogenase 1 which was associated with seed oil accumulation, expressed highly in seeds, and especially in high oil content material. The results and data analysed the target relationship between the miRNAs and genes, providing important information for the regulation of genes related to soybean quality traits.

## Supplementary Materials

The following are available online at https://www.mdpi.com/2073-4425/10/6/408/s1. Table S1: Soy ontology definition, Table S2: Global analysis of small RNA-seq data, Table S3: miRNAs and target genes, Table S4: Comparison of miRNA expression, Table S5: Differentially expressed miRNAs and genes, Figure S1: Construction of the chromosome segment substitution line (CSSL) population.
