# Supplementary figures and images for: Analysis of miRNAs Targeted Storage Regulatory Genes during Soybean Seed Development Based on Transcriptome Sequencing
Source: Genes (Basel). 2019 May 28;10(6):408. doi: 10.3390/genes10060408 (PMC6628032; doi:10.3390/genes10060408)

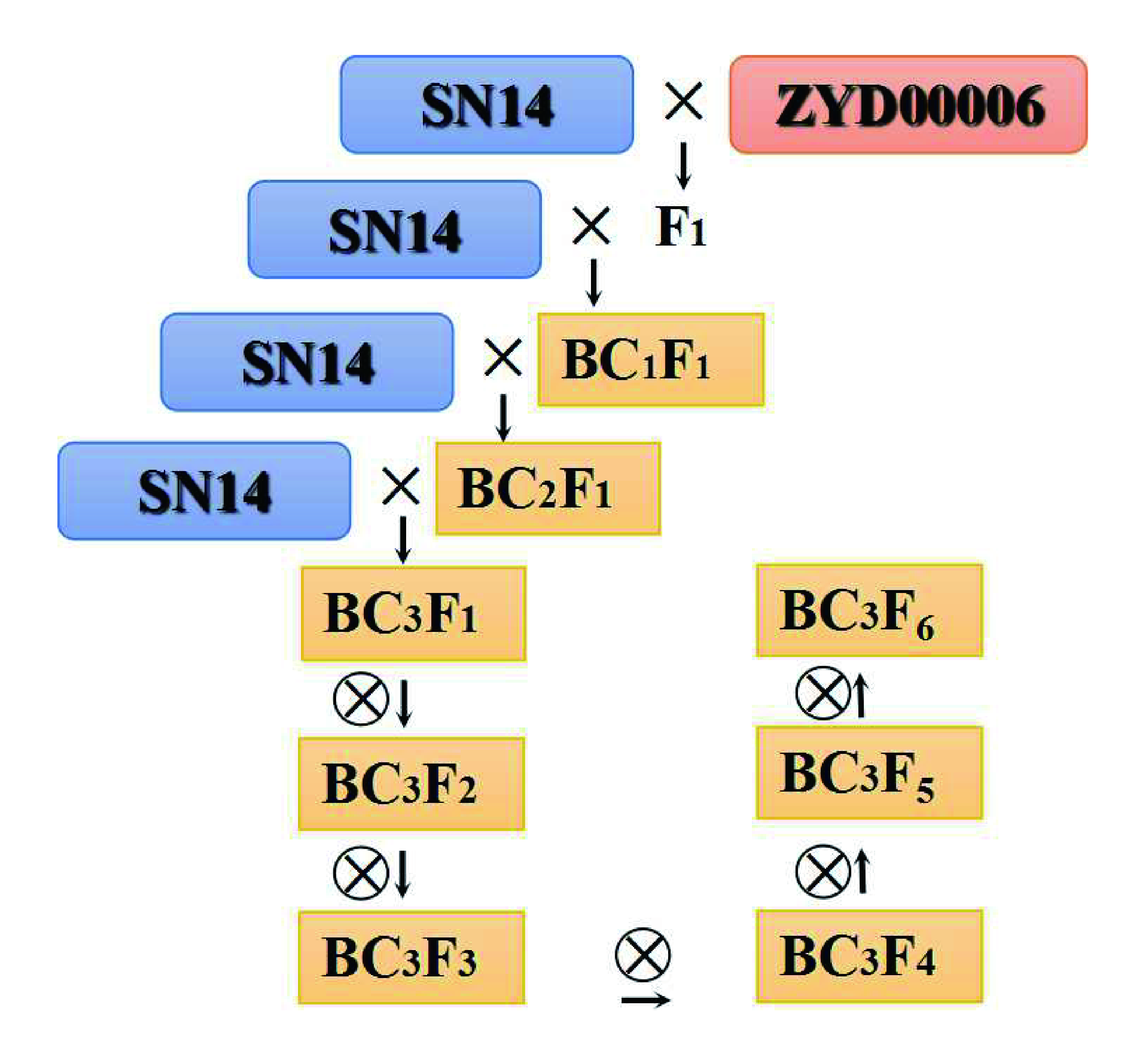

Supplement: Supplementary file 1 [file genes-10-00408-s001.zip › S1.jpg]
